# ShinyR-DAM: a program analyzing *Drosophila* activity, sleep and circadian rhythms

**DOI:** 10.1038/s42003-018-0031-9

**Published:** 2018-04-05

**Authors:** Karol Cichewicz, Jay Hirsh

**Affiliations:** Department of Biology, University of Virginia, Charlottesville, VA 22904 USA

## Abstract

We developed a web application ShinyR-DAM for analyzing *Drosophila* locomotor activity, sleep and circadian rhythms recorded by the *Drosophila* Activity Monitor (DAM) system (TriKinetics, Waltham, MA). Comparing with the existing programs for DAM data analysis, ShinyR-DAM greatly decreases the complexity and time required to analyze the data, producing informative and customizable plots, summary tables, and data files for statistical analysis. Our program has an intuitive graphical user interface that enables novice users to quickly perform complex analyses.

## Abstract (web summary)

Karol Cichewicz and Jay Hirsh developed a free, open-source, cloud-based application called ShinyR-DAM for analyzing *Drosophila* activity, sleep and circadian rhythms. ShinyR-DAM allows users to analyze their data quickly in customizable ways, improving the productivity of researchers.

## Introduction

The *Drosophila* Activity Monitor (DAM) system, (TriKinetics, Waltham, MA), is a set of devices for recording *Drosophila* locomotor behavior that is widely used around the world. The DAM system records infrared beam breaks of flies walking in glass tubes. Each DAM monitor has 32 channels recording locomotor behavior of individual flies. With many monitors used in an experiment, hundreds of individuals can be tracked simultaneously. The tubes housing flies during an experiment contain fly food, allowing monitoring of behavior over many days or even weeks, and the addition of drugs into the food allows modulation of fly physiology and behavior^1, 2^. During the course of an experiment, DAM monitors are housed in temperature, humidity and light controlled incubators, which allow study of fly environmental responses, as well as modulation their physiology using temperature dependent expression of transgenes^3–7^. Long-term data recording, and the capability to monitor behavior in darkness make this system particularly suitable for studying circadian rhythms^8^.

Currently available programs for DAM system data analysis (e.g., ClockLab, D. Ferster; pySolo^9^; ActogramJ^10^), require multiple steps of data processing, complex installation in specific computational environments, or expensive licenses. Additionally, most mandate manual detection of dead flies by tedious manual inspection.

With these limitations of existing programs in mind, we developed a new, free, open-source, cloud-based application, ShinyR-DAM **(**https://karolcichewicz.shinyapps.io/shinyr-dam/**)**. Our program is designed around a classic experimental circadian free run paradigm, in which animals are entrained to a 24 h cycle of a 12 h day and 12 h night (LD), followed by a period of constant darkness (DD), when animals express their free running circadian rhythm of sleep and activity. ShinyR-DAM can perform separate data analyses on LD vs. DD days, as well as within the day and night phases in LD. It allows rapid data analysis, producing customizable plots, and spreadsheet-readable comma-separated values (CSV) files. ShinyR-DAM does not require installation on a local computer, and it can be accessed from any modern internet browser. The name ShinyR-DAM, originates from the DAM system and the usage of Shiny framework^11^ for the R programming language^12^. R is one of the most popular programming languages among data scientists and biostatisticians, with broad spectrum of packages developed for life sciences, great plotting capabilities^13^, and supportive community of users. Because of the tremendous popularity of the R language among biologists, the ShinyR-DAM code is broadly accessible for editing and customizations in other laboratories.

## Results

**Fig. 1:**
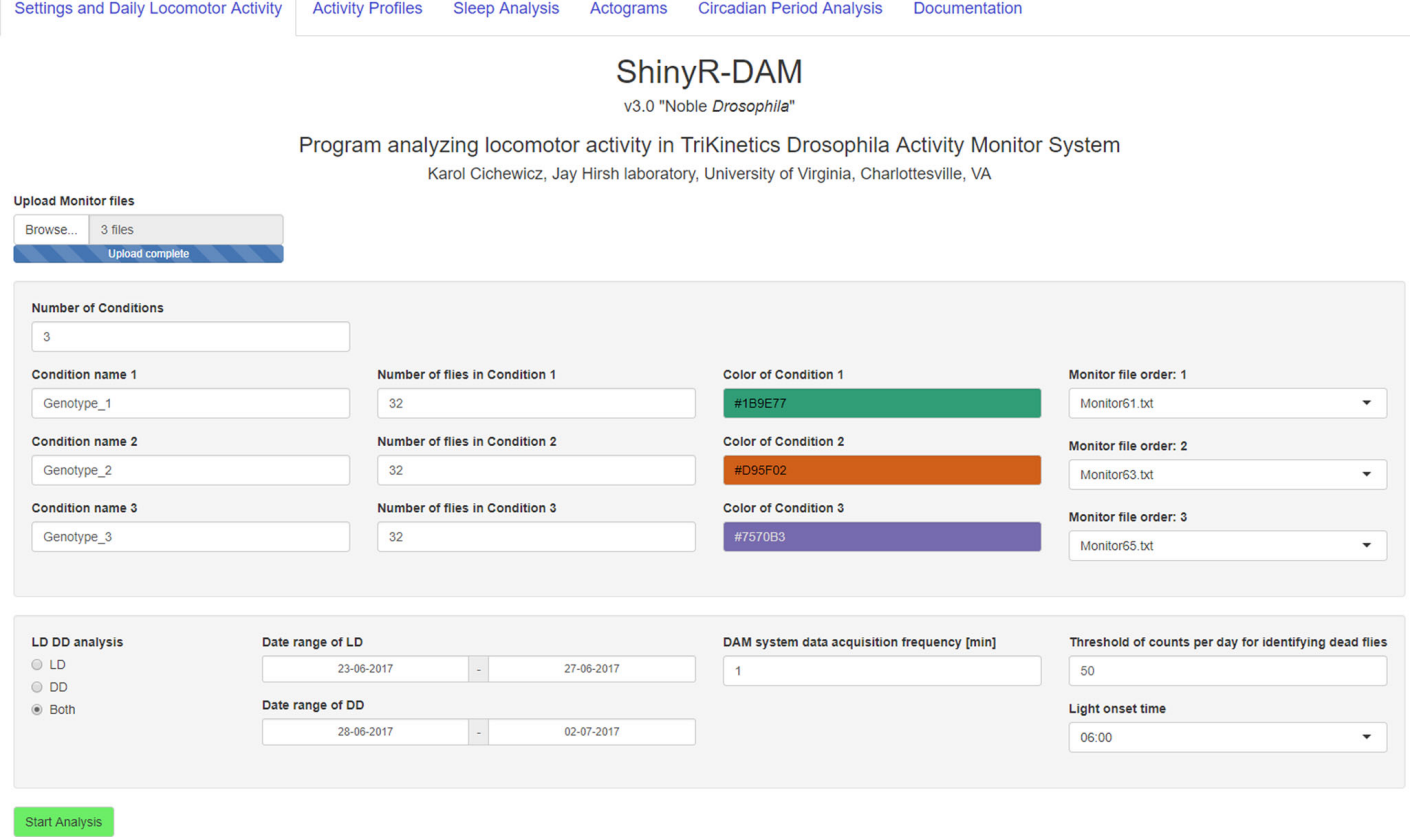
ShinyR-DAM user interface for uploading monitor files and specifying the experiment layout: number of conditions, condition names, number of flies in each condition, condition colors, and condition assignment to monitor files. The user can choose to analyze LD, DD, or both light regimes. The date range of LD vs. DD filters the experimental days from the monitor files. ‘DAM system data acquisition frequency’ allows ShinyR-DAM to analyze datasets that were recorded with any DAM system acquisition frequency. ‘Light onset time’ specifies when the day begins/night ends during LD. ‘Threshold of counts per day for identifying dead flies’ can be adjusted based on lab preference

ShinyR-DAM accepts DAM system monitor files recorded by the TriKinetics DAMSystem3 data acquisition software^14^. Users of the legacy DAMSystem2 data acquisition software can convert their channel files into monitor files using our file converter https://karolcichewicz.shinyapps.io/DAM2_to_DAM3_converter/. Using a graphical interface (Fig. 1), a user specifies the layout of the experimental conditions, including genotypes and treatments, and assigns them to specific monitors and channels. Each condition can have a user specified color for plot output. By default, each condition is assigned to a 32-channel DAM system monitor, but this setting can be overwritten to accommodate any experimental layout. ShinyR-DAM performs multiple tests for hardware and data integrity errors that may be recorded in monitor files, as well as tests for conflicts in the analysis settings. In case errors are detected, the user is informed about the nature of the problem and suggested solutions.

One major time saving feature of ShinyR-DAM is the automatic exclusion of dead flies. This feature eliminates tedious manual data inspection, and the often-arbitrary exclusion of inactive individuals. Dead flies are identified based on an adjustable daily locomotor activity threshold, and are excluded from analysis. Information about the number of dead flies in each condition is provided in a table.

**Fig. 2:**
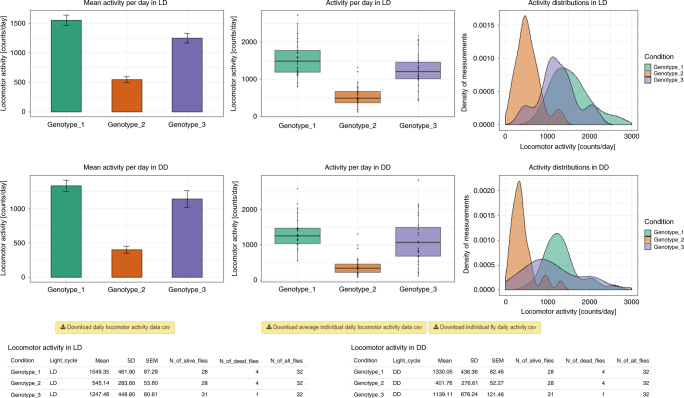
Locomotor activity analysis in LD and DD summarized in bar plots, box plots, density plots and tables. Users can define plot colors in the settings panel shown in Fig. 1. Box plots and density plots visualize the population structure. CSV files can be downloaded for further statistical analysis and data replotting. Error bars represent SEM

ShinyR-DAM generates summary bar plots of daily mean locomotor activity per condition, with error bars representing standard error of the mean (SEM) between individuals (Fig. 2). Box plots and density plots visualize the behavior of individuals of each condition, providing a representation of the population structure. Data visualized in bar plots are presented in tables that include the number of dead flies identified in each condition (Fig. 2). The locomotor activity by day plot illustrates day to day changes in behavior that may be caused by biological effects or by unwanted environmental variables.

**Fig. 3:**
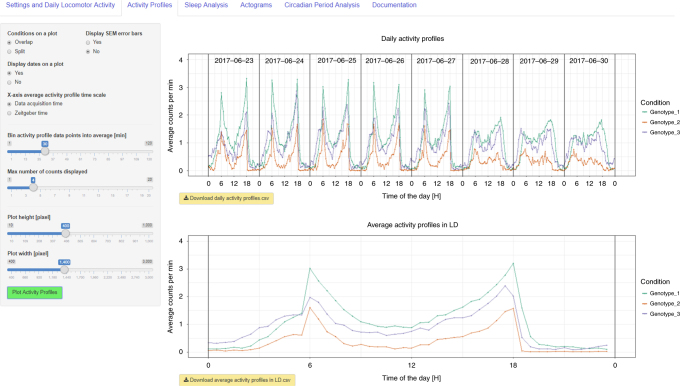
Activity profiles and user interface for adjusting the plots. ‘Daily activity profiles’ shows locomotor activity of each condition throughout the experiment. ‘Average activity in LD’ shows an average profile for all LD days. The user can choose to plot all experimental conditions in one plot, or split them into separate plots. SEM error bars and date annotations can be displayed or hidden. Data points can be binned into average values spanning an adjustable time window, which specifies the plot temporal resolution. The *Y* axis limit, the maximum number of counts displayed, plot height and width can be adjusted

Activity profiles (Fig. 3) show complete daily patterns of average locomotor activity of experimental conditions. A user can customize the plots by choosing to plot SEM error bars, split activity profiles of experimental conditions into individual plots, bin the displayed data, and graphically adjust the width and height of the plots. These customizations allow easy generation of publication-ready figures.

ShinyR-DAM calculates sleep using a standard sleep definition of continuous period of inactivity lasting at least 5 min. Using this definition, a sliding window algorithm detects sleep events in individual flies. Sleep data points are given values of 1 for sleep, and 0 for no-sleep, and are averaged over individuals in a condition. Sleep profiles offer similar customization options to activity profiles. Daytime and nighttime sleep per condition is presented in bar plots and tables. Average sleep and activity bout number and length are calculated and visualized in plots as well.

Actograms are the standard circadian plots displaying the activity time series data. ShinyR-DAM users can choose to plot mean, median, or individual single or double plotted actograms. A double plotted actogram displays two consecutive days in the same line, repeating the second day from the previous line in the successive line of the plot. This circadian plotting convention helps to visualize activity patterns that cross the midnight division.

ShinyR-DAM analyzes circadian periodicity in DD using the chi-square periodogram algorithm^15, 16^ adapted from the ‘xsp’ R package^17^. We customized the original periodogram function by allowing adjustments of temporal resolution of the algorithm and the range of tested periods. Our algorithm can also filter arrhythmic individuals based on a threshold of periodicity strength, calculated as a ratio of a periodogram peak value to its significance. Free run circadian periods and the period strengths are visualized in mean and individual periodograms, tables, and box plots.

ShinyR-DAM allows users to download data files that can be used for further statistical testing^14^. It produces esthetically satisfying plots, but specific journal guidelines may require further modifications or specific plot resolution and format. We provided R code snippets that will help users to recreate and edit ShinyR-DAM plots from the downloaded CSV data^14^.

ShinyR-DAM shines new light on analyzing *Drosophila* behavior recorded by the DAM system. It combines multiple powerful analytical algorithms, plotting functions and an intuitive graphical user interface that allow easy and deep exploration of the data. Compared to other software solutions for analyzing DAM data, ShinyR-DAM greatly improves productivity due to dramatic decreases in time required for data analyses.

### Data availability

ShinyR-DAM source code and test files are available through GitHub at: https://github.com/KarolCichewicz/ShinyR-DAM. ShinyR-DAM is implemented in R language and provided under the GPLv3 free software license. Source code of the Channel to Monitor file converter is available through GitHub at: https://github.com/KarolCichewicz/DAMSystem2-Channel-to-DAMSystem3-Monitor-File-Converter. Supplementary dataset for plotting scripts and a user manual are available at https://figshare.com/articles/ShinyR-DAM_Supplementary_Files/5858343.
